# A review on eye diseases induced by blue light: pathology, model, active ingredients and mechanisms

**DOI:** 10.3389/fphar.2025.1513406

**Published:** 2025-01-23

**Authors:** Yuan Yan, Yiyao Wu, Yu Zhao, Yaguang Yang, Guangtao An, Zhidong Liu, Dongli Qi

**Affiliations:** ^1^ Haihe Laboratory of Modern Chinese Medicine, Tianjin University of Traditional Chinese Medicine, Tianjin, China; ^2^ Engineering Research Center of Modern Chinese Medicine Discovery and Preparation Technique, Ministry of Education, Tianjin University of Traditional Chinese Medicine, Tianjin, China; ^3^ State Key Laboratory of Component-Based Chinese Medicine, Tianjin University of Traditional Chinese Medicine, Tianjin, China; ^4^ College of Chinese Medicine, Tianjin University of Chinese Medicine, Tianjin, China

**Keywords:** eye, blue light damage, pharmacological mechanism, modeling conditions, research direction

## Abstract

Blue light induced eye damage (BLED) belongs to modern diseases. It is an ophthalmic disease caused by prolonged exposure to electronic devices or screens containing a large amount of high-energy short waves (blue light). Specific symptoms include dryness and discomfort in the eyes, blurred vision, headache, insomnia, and in severe cases, it may also cause various eye diseases such as cataracts and glaucoma. At present, the development of health products and drugs for eye blue light injury faces many difficulties. Therefore, further exploration and research are needed on the pathogenesis, pathophysiology, and pharmacological mechanisms of blue light injury. Natural medicine ingredients and preparations have unique advantages in targeting eye blue light injury fatigue products due to their multi-component synergistic effects, overall regulation, and mild and safe characteristics. Starting from the disease-related mechanisms and pathophysiological characteristics of eye blue light injury, this article elucidates the pharmacological mechanisms of various drugs for treating eye blue light injury. At the same time, it reviews the research on *in vitro* cultured cell and animal model conditions for blue light injury eyes, in order to provide reference for subsequent blue light injury modeling experiments. And explore future research directions to provide new ideas and methods for the prevention and treatment of BLED.

## Graphical Abstract

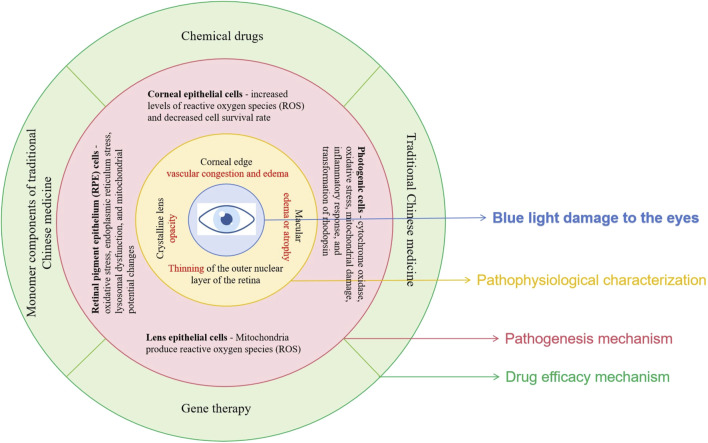

## 1 Introduction

In recent years, with the popularity of electronic devices such as computers and smartphones, people are increasingly exposed to artificial light sources (Ouyang et al., 2020), and prolonged exposure to light has become a major challenge affecting public visual health. The phototoxicity of visible light, especially blue light, has been extensively studied. Research has shown that short wave blue light between 400 and 460 nm is the most harmful (Wang and Li, 2021), as it can induce symptoms such as eye fatigue, dryness, pain, blurred vision, headache, insomnia, etc. Because it can penetrate the cornea and lens, directly reaching the retina, causing irreversible photochemical damage, known as blue light hazard (Kara-Junior et al., 2011; van Norren and Vos, 2016). However, endogenous pretreatment can to some extent alleviate the damage of blue light to biological tissues. It is a natural protective mechanism in organisms, which can reduce the damage of harmful light exposure to photoreceptors and RPE cells by synthesizing photoprotective pigments (such as melanin and lipofuscin) to absorb blue light, activate antioxidant enzymes, neutralize free radicals, etc. The subthreshold micropulse yellow laser (SMYL) treatment effectively treats macular edema by stimulating retinal pigment epithelial (RPE) cells with light, reducing the number of inflammatory cytokines and hyper-reflective retinal spots (HRS) (Bonfiglio et al., 2022).

Blue light damage to the eyes has led to an increase in various ophthalmic diseases such as age-related macular degeneration (AMD) (Jin and Jeong, 2022; Xia et al., 2019), cataracts (Xiang et al., 2020), and keratitis (Li et al., 2020). Epidemiological studies have shown that AMD is a human retinal degenerative disease affecting people aged 55 and above in industrialized countries, and is the main cause of blindness. Studies have shown that long-term accumulation of light exposure (especially blue light) can lead to retinal degeneration, causing damage to RPE cells and photoreceptor cells. AMD is mainly caused by functional impairment and loss of RPE, indicating that blue light is involved in the generation and development of AMD. Among patients aged 75 and above, the risk of early AMD is 25%, and the risk of late AMD is 8%. As the population ages, the absolute number of AMD patients worldwide will increase (Thomas et al., 2021). It is expected that by 2040, there will be 288 million AMD patients worldwide, with Asia having the highest number of disease patients (Keenan et al., 2021).

Many ophthalmic diseases caused by blue light damage can seriously affect people’s quality of life. To further investigate the pathogenesis and treatment methods of blue light damage, Based on the pathogenesis and pathophysiology of eye blue light injury, this article summarizes the pharmacological mechanisms of chemical small molecule drugs, natural drugs, and gene drugs related to the treatment of eye blue light injury. And summarize and organize the modeling conditions of cells and animals damaged by blue light, providing reference for subsequent blue light damage modeling experiments. Simultaneously exploring future research directions in order to lay the foundation for the application and development of natural medicine in the treatment of eye diseases.

## 2 Pathological characteristics and pathogenesis of blue light damage in the eyes

### 2.1 Pathophysiological characterization

The pathways of blue light damage mainly include oxidative stress, endoplasmic reticulum stress and oxidative DNA damage, inflammatory response, mitochondrial damage and cell apoptosis, lysosomal autophagy and vascular endothelial damage (Fan et al., 2022; Zhang H. et al., 2023). On the ocular surface, blue light irradiation can induce disordered autophagy levels in corneal stromal cells, affecting ocular surface function (Niwano et al., 2014). High energy and prolonged exposure to blue light can also penetrate the cornea and enter the lens, and the pathogenesis of cataracts may be closely related to oxidative damage to lens epithelial cells (Wang and Li, 2021; Liu, 2019). Blue light enters the retina, stimulating photoreceptors in the retina and photosensitive pigments in pigment epithelial cells, such as rhodopsin in photoreceptors and lipofuscin in pigment epithelial cells, producing a large amount of free radicals and reactive oxygen species (ROS). Lipid peroxidation is caused by ROS, which can lead to oxidative stress. Oxidative stress and photochemical damage can activate the apoptotic signaling pathway within cells, resulting in programmed cell death (Nan et al., 2023; Marie et al., 2018). In addition, the fluorescent group A2E of lipofuscin is activated by blue light, releasing free radicals and causing lipid peroxidation. This not only activates inflammatory reactions but also causes DNA breakage, while inhibiting the normal function of mitochondria and lysosomes (Moreira and Oliveira, 2011; Davies et al., 2001; Alaimo et al., 2019).

**FIGURE 1:**
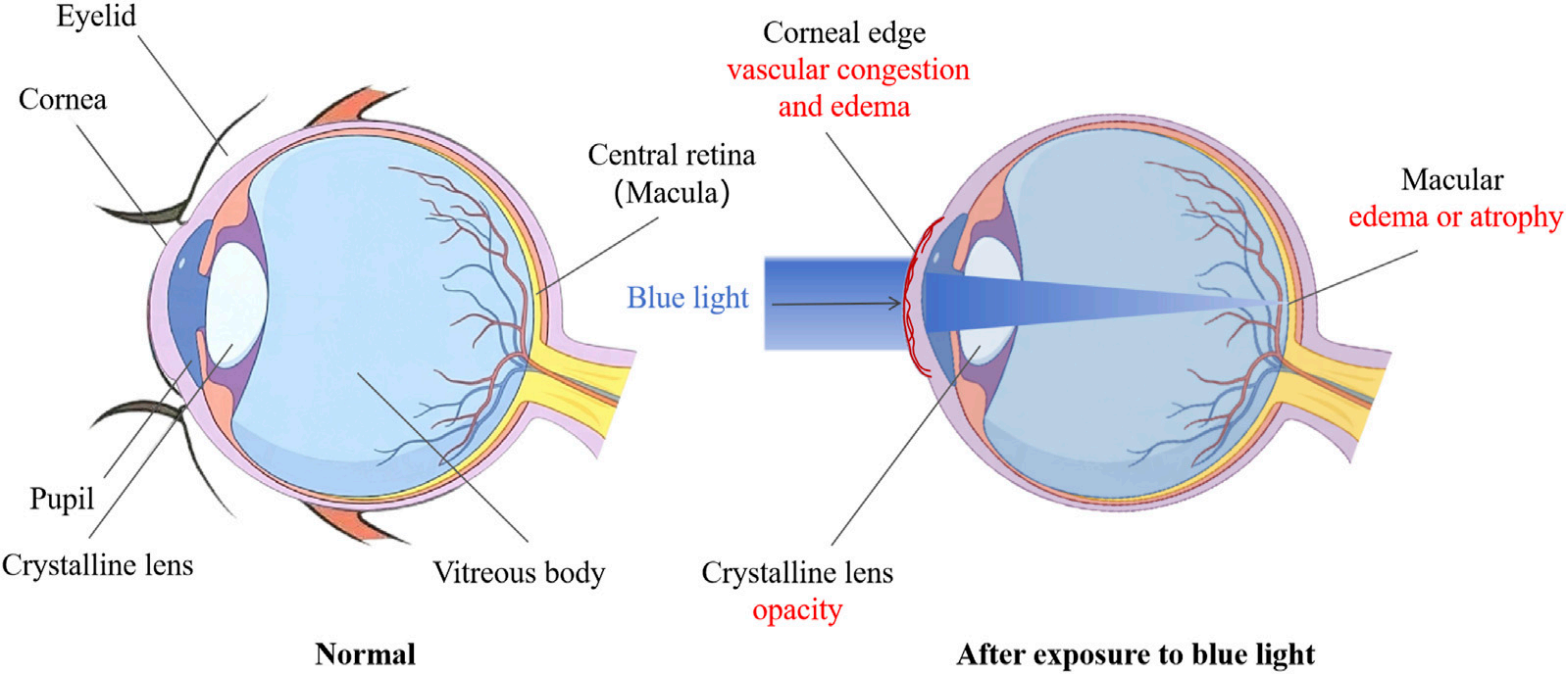
Changes in eyeball structure before and after blue light.

According to morphological observations, blue light irradiation can cause congestion and edema of corneal limbal blood vessels, dilation and thinning of the central part of the cornea, protrusion forward, and increased curvature. Long term exposure to blue light can cause the lens to become opaque, and the degree of opacity of the lens increases with prolonged exposure time. After blue light injury, the outer nuclear layer of the retina significantly thins, and due to degeneration and necrosis of some cells, the loss of outer nuclear layer cells decreases. Because ONL belongs to the photoreceptor cell layer, it often experiences a decrease in thickness and a decrease in the number of rod and cone cells when damaged, leading to problems such as decreased visual sensitivity, loss of field of view, and color vision abnormalities (Choi et al., 2016). Due to the release of pro-inflammatory cytokines, the permeability of blood vessels increases, and some harmful components in the blood can seep into the retina, leading to partial cell apoptosis. In addition, nuclear condensation and some irregular nuclei may occur, and fragmented nuclei can be seen (Shang et al., 2014). The pathological changes of blue light damage to the ocular surface, crystalline lens, and retina mentioned above may lead to various eye diseases, such as keratitis, dry eye syndrome, cataracts, myopia, and age-related macular degeneration (Figure 1).

### 2.2 Pathogenesis

#### 2.2.1 Effects of blue light on cornea and lens

Research has shown that blue light toxicity is not limited to the retina, but can also damage the ocular surface through oxidative stress, inflammatory response, and cell apoptosis, and is associated with the formation of ocular surface inflammation and dry eye syndrome (Lee et al., 2016; Chong, 2022). High energy blue light is close to ultraviolet light in the spectrum, so excessive exposure to blue light poses a great risk to the ocular surface. Blue light has high energy and can penetrate the cornea and lens to reach the retina. Due to its location in the anterior part of the eyeball, the cornea is the first point of contact for light entering the eye (Zhao et al., 2018). Therefore, blue light can directly act on corneal epithelial cells and endothelial cells, leading to a decrease in cell survival rate (Marek et al., 2018). At the same time, the content of reactive oxygen species (ROS) and the secretion of interleukin IL-1 β in the cornea will also increase, further mediating cellular oxidative damage and inflammatory response. The transparency of the crystalline lens decreases with age, leading to a gradual increase in absorbance within the blue light spectrum (Lee et al., 2016). Certain structural proteins, protein metabolites, and enzymes in the crystalline lens can absorb blue light, which can induce the production of reactive oxygen species (ROS) in the mitochondria of lens epithelial cells and lead to cell apoptosis through the transforming growth factor - β/Smad3 signaling pathway. This can lead to the gradual darkening and yellowing of the crystalline lens, which in turn can trigger cataracts (Cougnard-Gregoire et al., 2023). In addition, blue light damage can also trigger inflammatory reactions on the ocular surface, with damaged cells releasing inflammatory factors that attract the infiltration of inflammatory cells, further exacerbating tissue damage (Ouyang et al., 2023).

#### 2.2.2 Effects of blue light on the retina

Research has shown that the mechanism of retinal blue light damage is mainly related to the production of free radicals, lipid peroxidation, lipofuscin, rhodopsin, Ca^2+^levels, and other factors. The main ones involved are RPE cells and photoreceptor cells (Qin et al., 2019). Short term (minutes to hours) exposure to high irradiance blue light can cause damage to RPE cells (Tosini et al., 2016). Lipofuscin accumulates extensively in RPE cells, and its toxicity is determined by the total content of its main fluorescent group, N-subretinoyl-N-retinol-ethanolamine (A2E). Blue light can accelerate the oxidation of cells by A2E, leading to oxidative stress response and inducing RPE cell apoptosis. Researchers can search for effective candidate drugs that may treat blue light damage based on this mechanism (Conti et al., 2021). Lipofuscin generates a large amount of ROS under blue light irradiation, which triggers endoplasmic reticulum stress (ER stress) in RPE cells, manifested by upregulation of GRP 78 and CHOP protein expression, and then induces cell apoptosis through activation of Caspase-3 and decreased expression of Bcl-2 (Zhao, 2015). Blue light irradiation leads to dysfunction of lysosomes, decreased ability of lysosome phagocytosis and autophagy, accumulation of lipofuscin in cells, disruption of epithelial barrier, and ultimately resulting in cell apoptosis; Blue light can also affect the intracellular Ca^2+^concentration in RPE cells, causing changes in mitochondrial transmembrane potential and leading to cell apoptosis (Yang et al., 2023). In addition, blue light irradiation can also cause damage to retinal vascular endothelium. Hypoxia inducible factor 1 α (HIF-1 α) is significantly expressed in RPE, and as the main hypoxia sensor, it can regulate the expression of vascular endothelial growth factor (VEGF). VEGF plays a key role in angiogenesis and vascular permeability, but its overexpression can damage endothelial function and epithelial mesenchymal transition (Zhang Y. et al., 2023). Research has found that the expression of HIF-1 α and VEGF is elevated in the retina of rabbits with light damage, indicating that light exposure can induce endothelial dysfunction in the retina (Wang et al., 2016). Therefore, the mechanism of blue light induced RPE cell apoptosis is related to oxidative stress, endoplasmic reticulum stress, lysosomal dysfunction, and mitochondrial potential changes.

**FIGURE 2:**
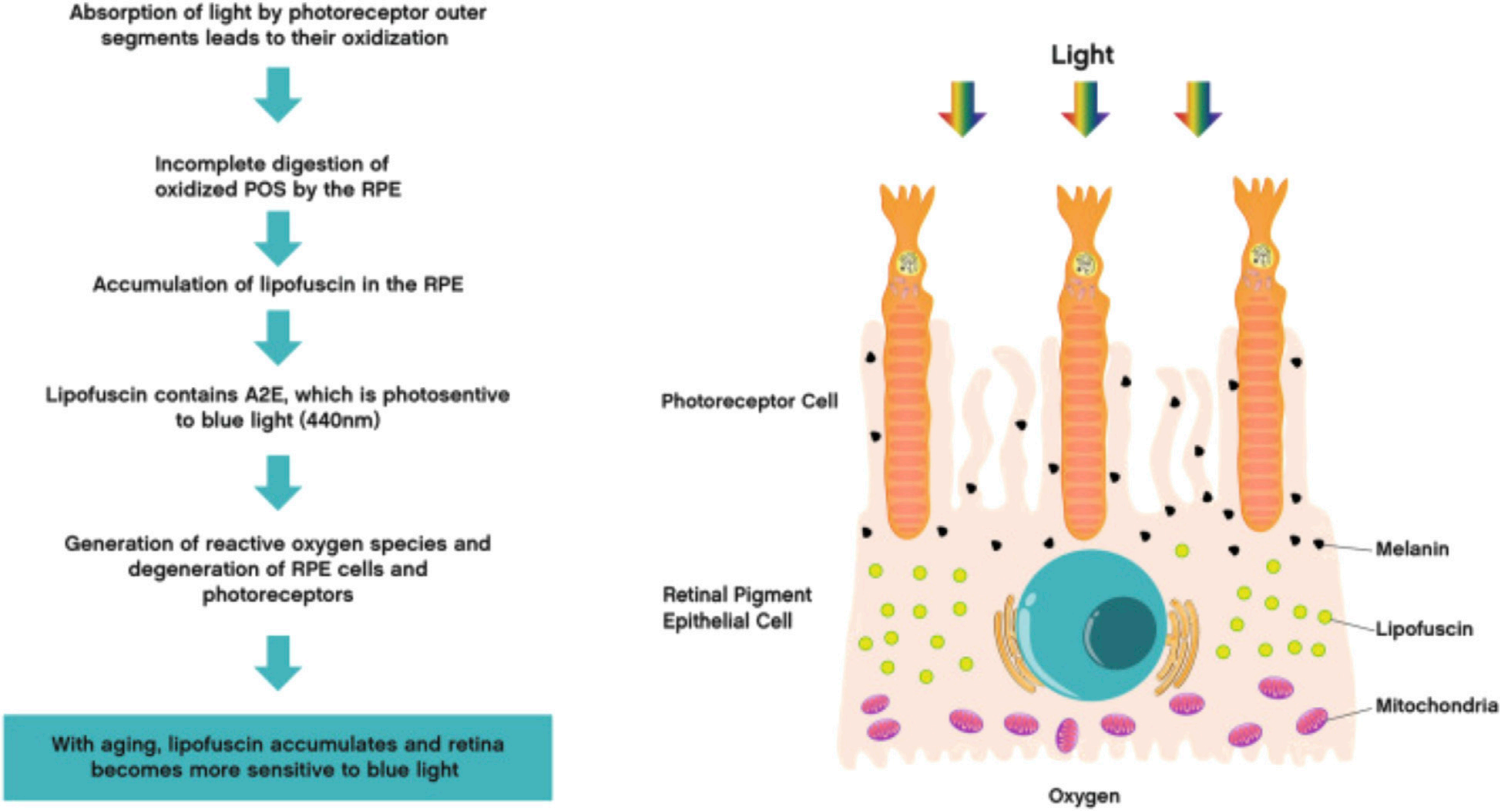
Mechanism of retinal blue light damage (Cougnard-Gregoire et al., 2023) (Quoted from Ophthalmology and therapy Journal).

Long term (several days to several weeks) exposure to low irradiance blue light can affect the wavelength activated photoreceptors (Tosini et al., 2016). High intensity blue light can cause irreversible inhibition of cytochrome oxidase, damage mitochondrial function and trigger cell apoptosis, while reducing sodium potassium ATPase activity, causing ion imbalance and increased osmotic pressure inside and outside the cell, leading to cell edema and organic damage, ultimately causing photoreceptor degeneration (Osborne et al., 2017). The degree of damage to photoreceptor cells (including cone cells and rod cells) exposed to blue light is higher, mainly due to the upregulation of retinal reactive oxygen species (ROS) production by blue light, which leads to mitochondrial damage in photoreceptor cells through oxidative stress. ROS induces the activation of mitogen activated kinase (MAPK), downregulates the phosphorylation level of extracellular regulatory protein kinase (p-ERK), upregulates activated nuclear transcription factor (NF-KB), activates autophagy pathway, and leads to apoptosis of retinal photoreceptor cells. After exposure to light, the expression level of pro-inflammatory factor miR-155 increases in the retina, while the expression level of anti-inflammatory factor SHIP1 decreases, leading to degeneration of retinal photoreceptors. Activation of NLRP3 inflammasome mediates the production of caspase-1 and 1L-1 β, leading to damage to retinal photoreceptor cells (Qin et al., 2019). Due to the presence of rhodopsin in rod cells and S-opsin in cone cells (Wheway et al., 2019), the conversion of rhodopsin by blue light can cause damage to both cone and rod cells, leading to blurred vision in the human eye. Due to the presence of rhodopsin, the photon capture ability of the retina is significantly enhanced under blue light irradiation, leading to an increase in the number of light induced cell deaths. Short wavelength LED light causes aggregation of short wavelength visual proteins, leading to degeneration of cone cells in the short term and severe damage to photoreceptor cells (Nakanishi et al., 2013). Blue light stimulation can activate the activity of prostaglandin synthase G/H. Prostaglandin synthase G/H is located in the inner and outer segments of rod and cone cells, promoting prostaglandin synthesis. It acts as a pigment group to absorb blue light, triggering oxidative reactions and producing a large amount of oxygen free radicals, leading to retinal damage and cell apoptosis (Xu and Wang, 2007). Therefore, the damage mechanism of blue light on photoreceptors is related to cytochrome oxidase, oxidative stress, mitochondrial damage, inflammatory response, and conversion of rhodopsin (Figure 2).

## 3 Common models for research on blue light damage to eye diseases

### 3.1 Summary of cell modeling conditions

The replication of disease models through *ex vivo* cells has the advantages of short experimental time, convenient operation, easy observation, good controllability, and reduced use of experimental animals. Currently, most studies on the mechanism of blue light induced retinopathy and drug anti blue light activity also use *in vitro* experiments. Therefore, selecting a suitable cell model is crucial.

The retina plays an important role in visual transduction, with retinal photoreceptors and RPE cells involved in visual formation. Photoreceptors are divided into rod cells and cone cells. Rod cells contain rhodopsin, while cone cells contain opsin (Wheway et al., 2019). When exposed to blue light, both rhodopsin and opsin undergo varying degrees of damage, leading to retinal degeneration. RPE cells are crucial for vision, maintaining the vitality and function of photoreceptors by engulfing detached outer segments of photoreceptors (Hellinen et al., 2019). In addition, they have multiple functions such as supporting the neuroretina and choroid in the eye, as well as immune suppression (Sugita et al., 2021), and are commonly used in ophthalmic research and general epithelial cell research (Hazim et al., 2019).

**FIGURE 3:**
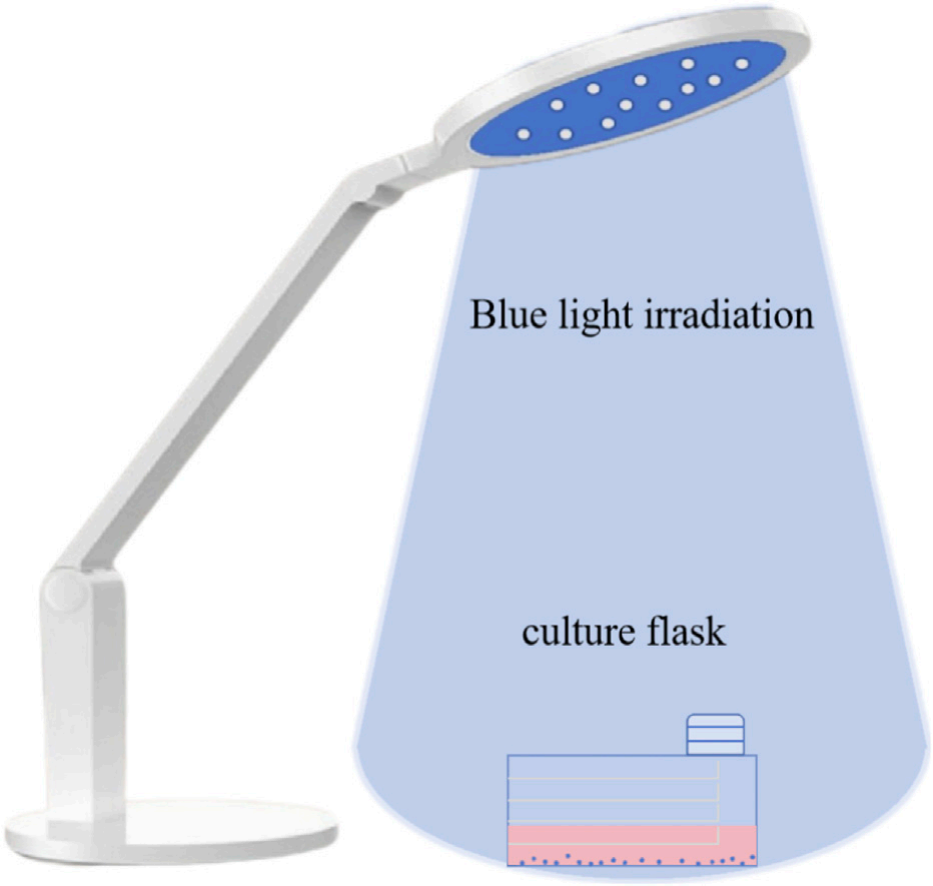
Schematic diagram of cell modeling.

Based on the physiological structure of the retina and the characteristics of blue light induced retinal lesions, mouse retinal photoreceptor cells (661W cells) and human retinal pigment epithelial cells (ARPE-19 cells) are commonly used cell models for light damage (Figure 3).

#### 3.1.1 661W cell model

661W cells are derived from retinal tumors formed in transgenic mice expressing SV40 large T antigen under the control of the IRBP (photoreceptor retinol binding protein) promoter. They are currently widely used in the study of glaucoma (Sayyad et al., 2017), macular degeneration (Huang et al., 2022), intraretinal neurodegeneration (Kunimi et al., 2021), and blue light induced retinal lesions. A study used a 661W cell model to investigate the improvement of blue light induced subcellular damage by swiftgrass extract. After drug pretreatment for 1 h, 661W cells were exposed to blue light with a wavelength of 464 nm and a light intensity of 0.38 mW/cm2 for 24 h. It was found that swiftgrass extract protected cells from blue light induced cell death by inhibiting the increase in ROS production, and had a protective effect on mitochondria and lysosomes (Yamazaki et al., 2024); At the same time, ARPE-19 cells and 661W cells were used to investigate the anti blue light activity of Dendrobium polysaccharides. The modeling conditions were irradiated with 2500lux light for 3 h, 6 h, and 16 h. After 6 h of illumination, there was a significant difference in cell survival rate between the model group and the control group, indicating successful modeling (Hsu et al., 2024); The anti blue light activity of astaxanthin was investigated using a 661W cell model. LED blue light tubes with a wavelength of 450 ± 20 nm were selected and placed in an incubator. After pretreatment with astaxanthin at different concentrations for 1 h, the cells were exposed to a light intensity of 2000lux for 24 h. After modeling was completed, the cells were further cultured in a complete medium containing the drug for 24–48 h, and the anti blue light activity of astaxanthin was evaluated by measuring ROS levels and mitochondrial damage degree (Lin et al., 2020).

#### 3.1.2 ARPE-19 cell model

The ARPE-19 cell line is derived from adult retinal pigment epithelial (RPE) explants, and ARPE-19 cells can be extensively expanded, providing a relatively stable source of retinal epithelial cells for functional and genetic research (Golconda et al., 2023). Lipofuscin is a byproduct of RPE cells engulfing the outer segment of photoreceptor cells, and its main fluorescent group is N-vinylidene retinyl-N-vinylethanolamine (A2E). When A2E is exposed to blue light, it is oxidized, inducing ROS production, oxidative stress, inflammatory response, etc., leading to RPE cell damage (Cho et al., 2023). Compared with 661W cells, ARPE-19 cells are more widely used in blue light damage research. Evaluate the anti blue light damage and retinal protective activity of the main active ingredient, hesperidin, in chrysanthemum using ARPE-19 cells. After pretreatment with A2E, expose the cells to blue light at a wavelength of 430 nm for 2 h, and the cell survival rate decreases by 50%, indicating the successful establishment of a light damage model (Feng et al., 2021); A study comparing two types of RPE cells, hTERT-RPE1 and ARPE-19, found that compared to hTERT-RPE1 cells, ARPE-19 cells have a stronger barrier function formed by tight junctions between cells. However, after being irradiated with blue light at a wavelength of 468 nm and a light intensity of 118.1 W/m^2^ for 4 h, the barrier function was impaired (Feng et al., 2021). The modeling conditions for the blue light damaged cell model are shown in (Table 1).

**TABLE 1:** Summary of modeling conditions for ARPE-19 cell model with blue light damage.

Wavelength	Light intensity	Light time	Other conditions	Cell survival rate (%)	References
430 nm	6000 lux	10–min	A2E pretreatment, drug pretreatment, and incubation for 6 h after modeling	47.8	Cho et al. (2023)
430 nm		2 h	A2E pretreatment, drug pretreatment	50	Feng et al. (2021)
468 nm	118.1 W/m^2^	4 h	-	Cellular barrier function impaired	Ozkaya et al. (2019)
450 nm	1500 lux	24 h	Drug pre protection	49.3 ± 10.5	Shimizu et al. (2022)
450 nm	2.3 mW/cm^2^	12 h	Drug pre protection	—	Wang et al. (2023)
430 nm	1000 lux	1 h	A2E pretreatment, drug pretreatment	—	Xie et al. (2021)

### 3.2 Summary of animal modeling conditions

Blue light damage animal modeling is the use of specific wavelengths of blue light to cause certain damage to animal eyes, in order to simulate the retinal damage that may occur in humans after prolonged exposure to blue light sources such as electronic screens. Its experimental technology is relatively mature and has a wide range of sources. The dorsal central region of the rat retina is considered a functional analogue of the human macular, which is the sharpest visual area (Gao et al., 2020). It has advantages such as simulation, controllability, repeatability, and safety. Therefore, through this model, researchers can better observe the impact of blue light on the visual system and explore methods for preventing or treating light damage, thereby providing scientific basis for protecting human visual health.

**FIGURE 4:**
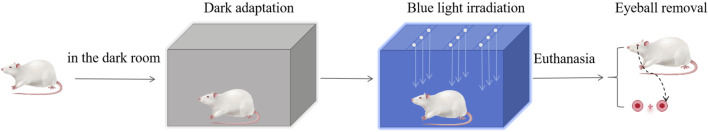
Schematic diagram of animal modeling.

The animal model of blue light damage is generally selected from 5–6 week old SD rats, which need to undergo 7 days of adaptive feeding and dark maintenance before officially starting modeling. Because dark maintenance can eliminate the effects of other light sources on rat eyes and increase the photosensitivity of rat eyes. The blue light wavelength selected for the experiment is generally concentrated around 450 nm, and the blue light irradiation time is mostly 3 h, 6 h, 8 h, 12 h. Among them, the 12 h light exposure/12 h dark cycle is used for the 12 h light exposure, and the duration of irradiation depends on the light intensity of the selected lamp source. It is more appropriate to choose a duration of 2 weeks (14 days) for light exposure modeling. Studies have shown that blue light illumination within the range of 2000–10000 lux can cause damage to the retina of rats (Shang et al., 2014). There is a certain conversion relationship between blue light illumination and blue light irradiance, for example, at 450 nm blue light, 1W/m^2^ = 28lux, and color temperature is also related to blue light irradiance. During experiments, a thermometer can be placed inside the cage to investigate the temperature inside the cage. If the temperature is too high, certain temperature dispersion measures need to be taken. At present, animal experiments on blue light induced damage in rats are mostly based on blue light intensity. The distance from the light source to the rat’s eyes needs to be determined by considering the actual height of the rat cage and the magnitude of blue light intensity at different distances (Figure 4).

In addition, if a positive drug group is required to evaluate drug efficacy, the blue light irradiation positive drug group generally needs to be administered orally 1 week before modeling and continue until the end of light exposure. The relevant inspection indicators are tested after the end of light exposure, and *in vivo* intraocular examination can choose slit lamp microscopy, fundus photography, and ERG detection. After the completion of the live examination, blood samples are usually collected and euthanized from the rats 24 h later, and the eyeballs are removed to prepare retinal sections. The examination indicators generally include HE staining, immunohistochemistry, Western blot detection, TUNEL assay, oxidative stress (ROS), and transmission electron microscopy (TEM) (Shang et al., 2014), in order to analyze the protective effect of drugs on the retina of rats with blue light injury. The modeling conditions for the blue light damaged cell model are shown in (Table 2).

**TABLE 2:** Summary of modeling conditions for blue light damage animal models (rats, rabbits).

Light source	Animal type	Wavelength	Duration of illumination	Number of days	Blue light illuminance	Blue light irradiance	Color temperature	Distance	References
OSRAM DULUXL BLUE LED Blue light	SD rat (5–6 weeks old)	480 nm	12 h	14 days	—	—	8000 k	10 cm	Chen (2021)
JDL Corporation (Hangzhou China)LED Blue light	SD rat	435–445 nm	8 h	10 days	31,360/44240lux	11.2 W/cm^2^	—	35 cm	Li et al. (2021b)
450 nm Semiconductor blue laser light source Zhuhai Aike Optoelectronics Technology Co., Ltd.	C57BL/6J mouse	450 nm	3 min irradiation/30 min rest-6 cycles,12 times	10 days	7.14/10.0725lux	2.55 mW/cm^2^ Intermittent low amplitude illumination	—	—	Zhang (2023a)
		450 nm	6 h	1 days	7.14/10.0725lux	2.55 mW/cm^2^ Continuous low amplitude illumination	—	—	
		450 nm	3 min irradiation/30 min rest-6 cycles,12 times	10 days	35.672/50.323lux	12.74 mW/cm^2^ High amplitude illumination interruption	—	—	
		450 nm	6 h	1 days	35.672/50.323lux	12.74 mW/cm^2^ Continuous high amplitude illumination	—	—	
BlueDog Technology Corporation Ltd.,Taibei, Taiwan	SD rat	460 nm	12 h	3, 9, 28 days	750lux	0.1 W/nm (power)	6500 k	20 cm	Zhang (2023b)
Blue light board (Zhongshan Gongxuan Optoelectronics Technology Co., Ltd.)	SD rat	455 nm	3 h	14 days	3,000 ± 50 lx	—	—	40 cm	Cheng et al. (2024)
Single wavelength LED Suzhou Jingzhi Medical Technology Co., Ltd.	SD rat	460 nm	10 min	1 month	1000 lux	—	—	15 cm	Zhu (2021)
		460 nm	10 min	1 month	2000 lux	—	—	15 cm	
Light damage tester (self-made)	SD rat	452 nm	1.5 h	1 days	1,400∼1 500 lux	—	—	—	Yang et al. (2020)
Zhongshan Gongxuan Optoelectronics Technology Co., Ltd.	BN rat	451 nm	3 h	1, 3, 7, 14 days	1,000 ± 100 lux	—	—	—	Yu et al. (2023a)
Lighting and Electromagnetic Department of the Center for Building Science and Technology (CSTB, Saint Martin d’here, France)	Wistar rat	449, 467, 473 nm	6, 12, 18, 24, 48, 72 h	7 days	—	0.0026 J/cm^2^(Ocean Optics- R2000+)	—	25 cm	Jaadane et al. (2015)
LED light strip (no manufacturer)	Wistar rat (12 weeks old)	463 ± 10 nm	12 h	10 days	150 lx	3.8 W/m^2^	—	—	Ziolkowska et al. (2023)
—	3-week-old rabbits (pigmented rabbits and albino rabbits)	450 ± 50 nm	12 h	21 days	420, 560, 1,120, 1820lux/592.5, 790, 1,580, 2567.5 lux	150, 200, 400 and 650 mW/cm^2^	—	—	Iseli et al. (2016)

## 4 Research progress on drugs for preventing and treating eye diseases caused by blue light damage

The drug therapy for anti blue light damage can be divided into chemical drugs, natural drugs, and gene drugs. At present, a series of research results have been achieved on how to alleviate the damage of blue light to eye cells, such as RPE cells and photoreceptor cells, through drugs. Based on the pathogenesis of blue light damage, anti blue light damage drugs can include free radical scavengers, antioxidants, anti-inflammatory drugs, and gene therapy (Pan et al., 2023). Oxidative stress plays an important role in the harm caused by blue light. Research has shown that effective antioxidant extracts related to eliminating free radicals reduce oxidative damage caused by blue light, thereby improving clinical symptoms of ocular surface in dry eye mouse models (Zhu et al., 2022). These three types of drugs each have their own advantages and limitations. The research and application of chemical drugs are relatively extensive, but there may be certain side effects. Natural medicines provide a more natural option, but their effectiveness and safety require further research to confirm. The application of gene therapy in eye diseases is an emerging field, but its technical complexity and potential risks also require further research and evaluation.

### 4.1 Medication routes and obstacles for the treatment of eye diseases

The anterior segment of the eye is composed of the cornea, conjunctiva, iris, ciliary body, crystalline lens, and aqueous humor, while the posterior segment of the eye includes the posterior two-thirds of the eye, including the vitreous, retina, choroid, and optic nerve (Vaneev et al., 2021). The blood-ocular barrier (BOB) includes the blood-aqueous barrier (BAB) located in the anterior segment of the eye and the blood-retinal barrier (BRB) located in the posterior segment of the eye, which can restrict the entry of drugs from the blood into the eye and is the main obstacle in treating eye diseases (Lyu et al., 2024). Drugs enter the body orally, are absorbed through the gastrointestinal tract, enter the liver through the portal vein, and some drugs may be metabolized by the liver or bind with plasma proteins before entering the systemic circulation and ultimately reaching the eyes. Among them, drugs need to overcome the first pass effect of the liver and the blood-ocular barrier to reach the eye, so their bioavailability is relatively low. The routes of drug delivery to the eye include local, periocular, intravitreal, suprachoroidal, and subretinal. Intravitreal, suprachoroidal, and subretinal administration is an ideal choice for treating posterior ocular lesions (Varela-Fernandez et al., 2020), as they can bypass more anterior barriers such as tear film, cornea, and sclera, but may cause injection related complications. External use mainly involves applying medication to the surface of the cornea or conjunctiva through eye drops or ointment. Eye surface administration is the most commonly used route of administration, but drugs are easily washed away by tears and difficult to penetrate the corneal barrier, so their therapeutic effect on posterior ocular diseases is limited. The development of new ocular drug delivery systems, such as nanoparticles, liposomes, and hyper choroidal injection (Singh et al., 2024; Wu et al., 2024), can effectively deliver drugs to the posterior segment of the eye (Puglia et al., 2021), which is expected to improve the ocular bioavailability and therapeutic efficacy of drugs.

### 4.2 The efficacy and mechanism of chemical drugs

At present, the research and application of chemical drugs for the treatment of BLED have become quite popular. The mechanism of action of these drugs is mainly achieved through antioxidant effects and the clearance of free radicals to achieve therapeutic effects. Therefore, anti blue light damage chemical drugs mainly include free radical scavengers and antioxidants. In addition, enzyme activity protectants, optic nerve protectants, calcium ion antagonists, and hormone drugs have also played a positive role in protecting retinal cells. The summary of the pharmacological mechanisms of chemical drugs for preventing blue light damage is shown in (Table 3).

**TABLE 3:** Summary of the pharmacological mechanisms of chemical drugs for preventing blue light damage.

Chemical compound	Type	Protection mechanism	Administration method	Dosage	References
Dimethyl thiourea (DMTU)	Free radical scavenger	Dimethylthiourea can reduce the content of malondialdehyde (MDA) in the retina of rats after photodamage, and has a protective effect on the retina	Rat Intravenous injection	50 mg/kg	Jin and Wan (2005)
Vitamin C	Free radical scavenger	Vitamin C has an anti lipid peroxidation effect and can significantly reduce the DNA damage of peripheral lymphocytes in healthy individuals caused by hydrogen peroxide. Vitamin C can increase the expression of Bcl-2 in retinal pigment epithelium after exposure to light, thereby inhibiting cell apoptosis	Rat Gavage method	100 mg/kg	Yin et al. (2011), Du et al. (2022)
Vitamin E	Antioxidant	Vitamin E can enhance the activity of intracellular SOD, resist lipid peroxidation, and reduce DNA damage of peripheral lymphocytes in healthy individuals caused by hydrogen peroxide,can protect photoreceptor cells	Human retinal pigment epithelium (hRPE) cells	10, 50, 100 μmol/L	Ueda et al. (2016), Wang et al. (2022)
Hydrogen sulfide	Antioxidant	H2S can clear intracellular ROS, increase SOD activity, and reduce intracellular oxidative stress and damage	Rat Sodium hydrosulfide as donor intraperitoneal injection	80, 120 μmol/kg	Zhu (2022)
Butylated hydroxytoluene (BHT)	Antioxidant	Butyl hydroxytoluene has the effect of inhibiting lipid peroxidation and malondialdehyde (MDA) production	—	—	Jiang (2002)
N-acetylcysteine (NAC)	Antioxidant	It can inhibit the production of ROS and the activation of NF - κ B, protect photoreceptor cells from damage caused by blue light, enhance cell viability, suppress cell death and erythropoietin, inhibit blue light induced caspase-3/7 activation and autophagy	Mouse cone photoreceptor derived cells (661 W)	—	Kuse et al. (2014)
Astaxanthin (AST)	Antioxidant	Astaxanthin can activate the Nrf2 signaling pathway, promote the expression of antioxidant enzymes and phase II detoxification genes, reduce intracellular ROS levels, alleviate mitochondrial damage, and ultimately protect ARPE-19 cells from oxidative stress damage induced by blue LED.	Mouse photoreceptor cells (661W)	1, 5, 10, 20 and 50 μM	Liu et al. (2021), Lai et al. (2020)
α-lipoic acid (ALA)	Antioxidant、Iron chelator	Alpha lipoic acid can inhibit iron mediated oxidative damage, suppress excessive iron accumulation, and may weaken retinal damage caused by oxidative stress through Nrf2 related endogenous antioxidant stress pathways. Lipoic acid can also counteract the accumulation of lipid peroxides inside the lens by increasing the activity of SOD and GSH Px, which may have a certain protective effect on photooxidative damage to the lens	ARPE-19 cells	150 μmol L^-1^	Yu et al. (2023b), Zhao et al. (2014)
Lipoic acid-niacin diad (N2L)	Antioxidant	N2L can increase the expression of various antioxidant proteins and counteract oxidative stress damage caused by blue light, which may be related to its upstream protein Nrf2/ARE pathway	Intraperitoneal injection	1.0, 2.5, 5.0 mg/kg	Cheng et al. (2024)
Fucoxanthin	Preventive antioxidant	Fucoidin has a significant improvement effect on visible light induced RPE cell phagocytic dysfunction by regulating the Nrf2 signaling pathway, and has good light absorption performance and antioxidant activity	-	0.1, 1, 10 mg/kg/d	Liu et al. (2016), Chen et al. (2021)
Lutein	Enzyme activity protectant	Participate in the formation of retinal macular pigment, quench oxygen free radicals, inhibit lipid peroxidation and the expression of c-fos gene to exert its protective effect	Rat Intravitreal injection Gavage method	0.5, 1.0 and 2.0 mg/mL; 25, 50, 100 mg/kg	Wang et al. (2008), Yang et al. (2020)
Zeaxanthin	Enzyme activity protectant	Blue light inhibits the activity of retinal cytochrome oxidase and sodium potassium ATPase, activates the activity of prostaglandin synthase G/H, and thus causes retinal damage	Mouse Gavage method	10 mg/kg/d	Sahin et al. (2019a), Ren et al. (2017)
Methoxyphenylpropionic Acid	Enzyme activity protectant	Non selective prostaglandin synthase G/H inhibitors can prevent a decrease in retinal ERGa and b-wave amplitude, protecting retinal function	—	—	Xu and Wang (2007)
Ciclofenaziae	Enzyme activity protectant	Non selective prostaglandin synthase G/H inhibitors can prevent a decrease in retinal ERGa and b-wave amplitude, protecting retinal function	—	—	Xu and Wang (2007)
Vitamin B1	Optic nerve protector	Vitamin B1 can reduce oxidative stress and protect cells from damage caused by free radicals; It can also protect the optic nerve from damage by enhancing the adaptability of nerve cells to energy demands	Oral administration	10 mg	Lakhan and Vieira (2010), Bai et al. (2023)
Methycobal	Optic nerve protector	Methycobal has a protective effect against glutamate induced neurotoxicity in retinal cells; It also has antioxidant and anti-inflammatory effects, helping to reduce oxidative stress and inflammatory reactions caused by photodamage	—	—	Kikuchi et al. (1997)
17-b estradiol	Optic nerve protector	Protect retinal mullium cells from H_2_O_2_ mediated oxidative damage, weaken H_2_O_2_ mediated cytotoxicity, and prevent light induced retinal degeneration and photoreceptor cell apoptosis	—	—	Shaban and Richter (2002)
Glutamate receptor antagonist MK-801	Optic nerve protector	It can reduce the proliferation of retinal pigment cells, alleviate laser damage to the retina, regulate the repair of retinal damage through glutamate, and has neuroprotective and anti proliferative effects on nerve cells	Rat Intramuscular injection	2 mg/kg	Yan (2006), Jiang (2002)
Flunarizine	Calcium antagonist	Flunarizine can prevent inositol triphosphate from releasing calcium ions into cells, showing sufficient protective effects on retinal pigment epithelial cells and photoreceptors	—	—	Xu (2008)
Flunarizine hydrochloride (FNZ)	Calcium antagonist	It can reduce the production of intracellular oxygen free radicals and stabilize the cell membrane	Rat Eye drops administration; Gavage method; Intravenous injection	Eye drops and gastric lavage group 14 mg/kg, Intravenous injection 5 mg/kg	Gong (2011), Dai (2020)
Taurine	Antioxidant、Calcium antagonist	Reduce intracellular calcium ion concentration to weaken calcium dependent Fas mediated apoptosis of neutrophils. And it can effectively eliminate free radicals, but it requires early administration	mouse; Intravenous injection	200 mg/kg	Gong (2011), Tao et al. (2019)
Glucocorticoid	Hormones	It can alleviate inflammatory reactions, maintain cell membrane structure, protect microcirculation, block lipid peroxidation, reduce the production and damage of free radicals, and have a preventive and therapeutic effect on retinal photodamage	Intravitreal injection	—	Feng (2012), Li and Tang (2023)
Estrogen	Hormones	Inhibit apoptosis of photoreceptor cells, inhibit the synthesis of alpha receptor nitric oxide synthase (NOS), reduce the production of nitric oxide, and alleviate retinal damage	Rat Hypodermic injection	50 μg/kg	Chen (2005), Xu (2008)

In addition, blue light damage is a cause of various eye diseases. Although current treatment methods are limited and mainly focus on relieving symptoms and delaying disease progression, researchers are actively exploring new treatment strategies. Research shows that both blue light damage and diabetes retinopathy (DR) involve the damage and dysfunction of retinal nerve cells. The locally administered NOX4 inhibitor GLX7013114 can effectively protect retinal neurons and amacrine cells, and alleviate oxidative stress and inflammatory reactions (Dionysopoulou et al., 2023). It may become a new drug for treating blue light damage. In addition to GLX7013114, lidocaine pretreatment has also been shown to prolong the lifespan and improve the function of retinal ganglion cells (RGCs) (Chou et al., 2018), which may provide another neuroprotective approach for treating blue light injury.

### 4.3 The efficacy and mechanism of natural medicine

**FIGURE 5:**
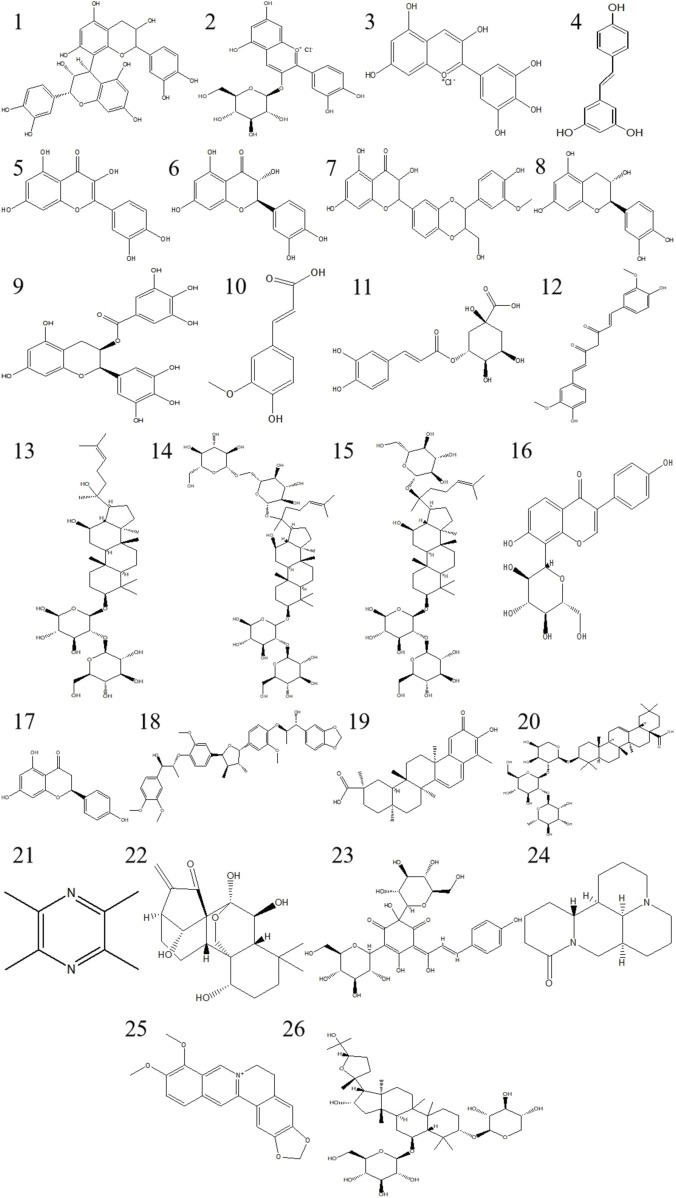
The compound structure of traditional Chinese medicine monomers includes:1 Procyanidin B2; 2 Cyanidin 3-O-glucoside chloride; 3 Delphinidin; 4 Resveratrol; 5 Quercetin; 6 Taxifolin; 7 Silymarin; 8 Catechin; 9 Epigallocatechin Gallate; 10 Ferulic acid; 11 Chlorogenic Acid; 12 Curcumin; 13 Ginsenoside Rg3; 14 Ginsenoside Rb1; 15 Ginsenoside Rd; 16 Puerarin; 17 Naringenin; 18 Manassantin B; 19 Celastrol; 20 Raddeanin A; 21 Tetramethylpyrazine; 22 Oridonin; 23 Hydroxysafflor Yellow A; 24 Matrine; 25 Berberine; 26 Astragaloside IV.

At present, research on natural medicines for combating blue light damage is mostly focused on antioxidants and anti-inflammatory drugs. For example, caffeine, as an approved drug for clinical use, has anti-inflammatory and neuroprotective effects, and its mechanism of action is oxidative stress and inflammatory response. Currently, studies have shown that caffeine may be a potential candidate for the treatment of retinal degeneration (Conti et al., 2021). The research results indicate that natural medicine has significant therapeutic effects in preventing and treating eye diseases caused by blue light (Li C. et al., 2021). Natural medicine, with its multi-component and multi-target characteristics, can play a role in different stages. It can not only effectively eliminate free radicals and reduce oxidative damage, but also inhibit inflammatory reactions and protect the integrity of eye cells. The following summarizes the individual components of natural medicine for preventing blue light damage and their pharmacological mechanisms, in order to provide reference for research and application in related fields (Figure 5). The summary of the pharmacological mechanisms of natural medicine monomers for preventing blue light damage is shown in (Table 4); The summary of the pharmacological mechanism of natural medicine for preventing blue light damage is shown in (Table 5).

**TABLE 4:** Summary of pharmacological mechanisms of monomers in natural medicine for preventing blue light damage.

Drug ingredients	Protection mechanism	Administration method	Dosage	References
Procyanidin B2	Procyanidin B2 inhibit blue light induced RPE cell damage by alleviating oxidative damage, endoplasmic reticulum stress response, and mitochondrial apoptosis pathways	ARPE-19 human retinal pigment epithelial cells	0.1, 0.5, 1, 5, 10 μmol/L	Xu et al. (2018), Zhao (2015)
Cyanidin 3-O-glucoside chloride (C3G)	C3G and its phenolic acid metabolites attenuate visible light induced retinal degeneration *in vivo* by activating the Nrf2/HO-1 pathway and inhibiting NF - κ B	Pigmented rabbit Oral administration	0.11 mmol/kg/d	Wang et al. (2016)
Delphinidin	Delphinidin can reduce the content of intracellular peroxide product TBARS after light damage, increase the activity of antioxidant enzyme systems (SOD, GSH Px, GST), and have a protective effect against light induced oxidative stress damage to the retina	661W photoreceptor cells	5, 10, 20 μmol/L	Peng et al. (2019)
Resveratrol	Upregulation of cell viability, SOD, CAT, GSH; downregulation of cell proliferation, ERK1/2 and MEK expression, as well as caspase-3, caspase-9,ROS,p-p38,p-ERK,p-JNK.	ARPE-19 cells	50, 100, 200 and 400 μmol/L	King et al. (2005), Ye and Meng (2021)
Quercetin	Upregulate cell viability, mitochondrial membrane potential, and phagocytic function; Downregulate the production of lipid hydroperoxides and singlet oxygen	ARPE-19 cells	25 μM, 50 μM and 100 μM	Olchawa et al. (2020)
Taxifolin	Taxifolin may protect the eyes from oxidative stress damage by regulating NRF2 levels and promoting phase II antioxidant enzyme activity	Rat Gavage method	10 mg/kg, 50 mg/kg	Zhang (2023c)
Silymarin	Silymarin can significantly protect RGCs from blue light damage by activating the MEK/ERK/CREB pathway, indicating that inflammatory factors may become targets for treating eye diseases	Retinal ganglion cells (RGCs)	50 μM and 100 μM	Shen et al. (2019)
Catechin	Catechin can significantly reduce the expression of NF - κ B and proinflammatory mediators (IL-1 β, IL-6, TNF - α) in diabetes retinopathy rats, so it can be used as an effective component in the treatment of diabetes retinopathy	Rat Intravitreal injection	50, 100, 200 mg/kg/d	Wang et al. (2018)
Epigallocatechin gallate (EGCG)	Upregulation of cell viability; Downregulate the expression of ROS, H_2_O_2_, p-JNK, p-ERK, p-p38, and COX-2; The expression of caspase-3 is not affected	Rat Oral administration	—	Chan et al. (2008), Zhang et al. (2008)
Ferulic Acid	Ferulic acid can alleviate cell damage induced by sodium iodate in retinal degeneration mice, and oral administration of ferulic acid can provide protective effects on the retina	Mouse Oral administration	10, 30 mg/kg/d	Kohno et al. (2020)
Chlorogenic acid	Chlorogenic acid has anti-inflammatory and antioxidant effects. Chlorogenic acid can activate the Nrf2 signaling pathway, promote Nrf2 nuclear entry and binding with ARE, thereby promoting the expression of phase II detoxifying enzymes (HO-1, NQO1, GCLC, and GCLM) and exerting antioxidant effects	ARPE-19 cells	25, 50, 100 μmol/L	Liu (2021)
Curcumin	Curcumin can protect RPE cells from blue light irradiation by reducing ROS levels and increasing VEGF, GSH, and GSH Px levels	ARPE-19 cells	20 μM	Bardak et al. (2017)
Ginsenoside Rg3	Ginsenoside Rg3 has various biological effects such as antioxidant and scavenging of oxygen free radicals. Inhibiting the expression of vascular endothelial growth factor (VEGF) has an inhibitory effect on the formation of corneal and choroidal neovascularization	Human umbilical vein endothelial cells HUVEC	100.0 μmol/L	Lu et al. (2017)
Ginsenoside Rb1 Ginsenoside Rd	The combined action of ginsenoside Rb1 and Rd (a saponin compound of Panax notoginseng saponins) can promote changes in the expression of miR-155 and SHIP1 in the retina and maintain them at normal levels, inhibiting light induced retinal degeneration	Mouse Intraperitoneal injection	Rb1 (65 mg/kg) and Rd (22.5 mg/kg)	Bian et al. (2017)
Puerarin	Puerarin has antioxidant and scavenging effects on oxygen free radicals in the body, which may reduce the production of advanced glycation end products (AGEs) and alleviate tissue damage caused by AGEs through antioxidant and lipid-lowering effects. It reduces the expression of hypoxia inducible factor (HIF-1 α) and the production of VEGF, thereby inhibiting the formation of new blood vessels in ischemic tissues	RPE cells	0.01, 0.1, 1 g/L	Nguyen et al. (2019), Li et al. (2006)
Naringenin	Naringin has various pharmacological activities such as anti-inflammatory and antioxidant effects, which can protect the retinal pigment epithelium and inhibit the formation of new blood vessels	Rat Eye drops administration	10 g/L 3 times	Shen et al. (2010a)
Manassantin B	Manassantin B has a protective effect against free radical damage, which can inhibit the generation of O_2_, antagonize the effects of OH and H_2_O_2_ on cell membrane lipid peroxidation, and enhance the activity of self antioxidant enzymes in the body	—	—	Jiang (2002)
Celastrol	Celastrol has anti-inflammatory, anti-tumor and immunosuppressive activities. Studies have shown that Triptolide can inhibit photoreceptor cell death, alleviate oxidative stress response of retinal pigment epithelium and photoreceptors, reduce the expression of pro-inflammatory genes in the retina, and inhibit the activation and gliosis of retinal microglia	Mouse Intraperitoneal injection	5 mg/kg	Yang et al. (2011), Bian et al. (2016)
Raddeanin A	Raddeanin A can inhibit oxygen induced retinal neovascularization, and its mechanism may be related to the inhibition of VEGFR2 mediated AKT and ERK1/2 signaling pathways	Mouse Intraperitoneal injection	180 mg/mL	Huang (2013)
Tetramethylpyrazine	Tetramethylpyrazine can inhibit the proliferation of endothelial cells and reverse hypoxia damage in human retinal pigment epithelial cells (ARPE-19). By inhibiting the expression of HIF-1 α in RPE cells induced by AGEs, the production of new blood vessels is suppressed	Rat Eye drops administration	25 μg 1%TMP eye drops	Shen et al. (2010b), Zou et al. (2007)
Oridonin	Research has shown that oridonin can effectively inhibit the proliferation, migration, and angiogenesis of monkey retinal endothelial cells, and suppress hypoxia induced retinal neovascularization in mice by blocking the focal adhesion kinase FAK/Paxillin signaling pathway	Mouse Intraperitoneal injection	—	Dong (2011)
Hydroxysafflor Yellow A	Hydroxysafflor Yellow A has pharmacological activities such as anti-inflammatory and antioxidant effects. Studies have shown that hydroxysafflower yellow pigment A can inhibit the proliferation of high glucose induced choroidal endothelial cells in rhesus monkeys, possibly by downregulating the expression of VEGF mRNA induced by high glucose	Rat Intraperitoneal injection	5.0 mg/kg	Fu et al. (2023)
Matrine	Matrine can effectively inhibit the proliferation of retinal microvascular endothelial cells (RRMECs) in rats and induce apoptosis of RRMECs, mainly by downregulating VEGF expression	Rat Gavage method	30, 60 and 90 mg/(kg·d)	Zhang (2019)
Berberine	The activation of the PI3K/AKT/ERK pathway can protect the retina from light induced degeneration, which may be related to reducing retinal oxidative stress	Mouse Gavage method	200 mg/kg/d	Song et al. (2016)
Astragaloside IV	Astragaloside IV upregulates the PINK1/Parkin signaling pathway in cells, inhibits the production of large amounts of ROS, avoids mitochondrial damage and apoptosis, and maintains cell viability and autophagy process	ARPE-19 cells	50 mg/L	Li et al. (2021c)

**TABLE 5:** Summary of the pharmacological mechanisms of traditional Chinese medicine for preventing blue light damage.

Drug ingredients	Protection mechanism	Administration method	Dosage	References
Wolfberry Extract	The polysaccharides in goji berries can significantly enhance the enzymatic antioxidant system enzyme activity, inhibit the production and accumulation of peroxidation product malondialdehyde, significantly reduce the body’s oxidation rate and degree, and alleviate oxidative stress damage to the eyes	Human retinal pigment epithelial cells (HRPE)	0.01, 0.1, 1 mg/mL	Dou (2011)
Dendrobium Officinale Extract	The polysaccharides in Dendrobium officinale can downregulate the levels of inflammatory factors and VEGF through the NF - κ B signaling pathway, inhibiting the inflammatory response and neovascularization in the retina of rats	Rat; Gavage method	100, 200, 300 mg/kg	Qin et al. (2021)
Blackcurrant Extract	Anthocyanins in blackcurrant can bind with free radicals under dehydrogenation, terminating the chain reaction of free radicals; It can also inhibit peroxidation through chelation reaction with metal catalysts	ARPE-19 cells	0.01, 0.10, 10.00 μg/mL	Wang et al. (2024a)
Blueberry Extract	Anthocyanins in blueberries can significantly reduce the levels of blood lactate and malondialdehyde, as well as significantly enhance the activity of superoxide dismutase and glutathione peroxidase, inhibit the large-scale production of downstream product NF kB, and achieve the effect of preventing oxidative damage	ARPE-19 cells	0.01, 0.10, 10.00 μg/mL	Wang et al. (2024b)
Cassia Seed Extract	Anthraquinones in Cassia seed can inhibit the activity of aldose reductase in ocular lens cells; Inhibit the generation of end products of oxidative glycosylation; Inhibiting oxidative DNA modification and nitroso tyrosine accumulation in retinal cells to achieve protective effects against oxidative stress damage in the eye	ARPE-19 cells	0.01, 0.10, 10.00 μg/mL	Wang et al. (2024a)
Chrysanthemum Extract	Osmanthus glycosides in chrysanthemums have the ability to scavenge DPPH free radicals *in vitro*, effectively alleviate oxidative stress in myocardial cells, improve amblyopia, dry eye syndrome, and macular function without altering retinal structure	HRPE-19 cells	25, 50, 100 μM	Yu (2019), Kan et al. (2020)
Bilberry Extract	Anthocyanins in bilberry can upregulate cell viability; Downregulate ROS generation, p38 MAPK phosphorylation, JNK phosphorylation	661W cells	1–30 μg/mL	Ogawa et al. (2013), Ogawa et al. (2014)
Palmleaf raspberry fruit	It has antioxidant effects on singlet oxygen by regulating the activation of NF - κ B, p38 MAPK, autophagy, and caspase-3/7 signaling pathways, inhibiting the production and activation of pro apoptotic proteins, and protecting retinal photoreceptors	Retinal ganglion cell (RGCs)	0.1, 1, 10, 25 μg/mL	Li (2017)
Marigold Extract	Lutein and zeaxanthin in marigold can upregulate antioxidant capacity, retinal Rho, Sag,Gnat1,NCAM,GAP43,BDNF,NGF,IGF1,Nrf2,HO-1; Downregulate the expression of NF kB and GFAP.	Rat; Gavage method	100 mg/kg	Sahin et al. (2019b), Wang et al. (2024a)

### 4.4 Efficacy mechanism of gene drug therapy

At present, some gene drugs can effectively treat BLED by enhancing cellular antioxidant capacity, regulating endoplasmic reticulum stress, and fundamentally reducing the damage of blue light to eye cells, thereby achieving good therapeutic effects. The mechanism of action of gene drugs is clear, and they can target eye cells with precise drug delivery and action, reducing side effects on other tissues and organs. The summary of the pharmacological mechanisms of gene therapy for preventing blue light damage is shown in (Table 6).

**TABLE 6:** Summary of pharmacological mechanisms of gene therapy for anti blue light damage.

Gene therapy	Protection mechanism	Administration method	Dosage	References
Nrf2 stress response transcription factor	Research has found that Nrf2 can to some extent protect photoreceptor cells from oxidative stress damage	661W cells Nrf2 knockdown	—	Chen et al. (2017)
Knocking out the cell surface chemokine receptor 2 (CCR2) gene	Research has found that knocking out the cell surface chemokine receptor 2 (CCR2) gene can significantly alleviate the death of mouse retinal photoreceptor cells caused by chronic blue light irradiation, while also inhibiting the activation and proliferation of microglia during light induced retinal degeneration	661W cells; Knock out CCR2	—	Hu et al. (2016)
Leukemia inhibitory factor (LIF)	Leukemia inhibitory factor (LIF) has an inhibitory effect on photodamage in retinal photoreceptor cells, possibly by activating the JAK3/STAT3 signaling channel to suppress downstream Bax/Bcl-2 apoptotic channels	Mouse; Light pre adaptation	—	Dong et al. (2018)
Extracellular vesicles derived from bone marrow mesenchymal stem cells (MSC-Exos)	By downregulating vascular endothelial growth factor-A (VEGF-A), we aim to improve the blue light stimulation and laser-induced retinal damage in retinal pigment epithelial cells (RPE cells)	Rat; Transplant	1.0, 2.0, 3.0 µL (50 μg/mL)	He et al. (2018)
Tissue factor targeting peptide (TF-TP)	Pre treatment of RPE cells with tissue factor targeting peptide (TF-TP) can reduce blue light damage and increase cell survival rate. Its mechanism of action is related to the inhibition of the Bax/Bcl-2 apoptosis pathway by TF-TP.	RPE cells	150 μmol/L	Li et al. (2017)
Heat shock protein 5(HSPA5)	HSPA5 has the ability to regulate endoplasmic reticulum stress, alleviate A2E and blue light damage, and promote RPE cell survival	RPE cells Transfection of HSPA5 interference series	—	Feng et al. (2019)
Estrogen related receptor α(ERRα)	Under blue light induction conditions, ERR α may exert anti apoptotic effects by promoting Bcl-2 expression in ARPE-19 cells	ARPE-19 cells Overexpression of ERR α	—	Lin, 2020; An (2022)
Glutaredoxin 2 (Grx2)	Grx2 may exert antioxidant stress effects by inhibiting the JNK signaling pathway, reducing cell apoptosis, and thereby alleviating the damage of blue light to the retina	Mouse; Knockout and Knockin of Grx2 Gene	—	Bin (2021)
Ceramide like protein (CERKL)	CERKL can alleviate blue light induced oxidative stress damage in ARPE-19 cells by activating SIRT1 protein expression and promoting deacetylation of E2F1	SiRNA CERKL and pcDNA3.1-CERKL transfection into ARPE-19 cells	—	Zhuang et al. (2022)
miRNA (miR-22-3p)	Overexpression of miR-22-3p can inhibit RGC apoptosis by suppressing PTEN, activating the PI3K/Akt/Nrf2 pathway, and thereby suppressing RGC apoptosis	Rat; Intravitreal injection	1 μL	Zhang et al. (2022)
Methyltransferase like protein (METTL7B)	Under blue light induction, METTL7B may exert a protective effect on RPE cells after light injury by promoting HO-1 expression in ARPE-19 cells; METTL7B may control ARPE-19 cell apoptosis through the BCL-2/BAX pathway by promoting BCL-2 and reducing BAX protein accumulation	ARPE-19 cells; Overexpression of METTL7B	—	Wang (2023)
Poly (ADP ribose) polymerase (PARP-1)	PARP-1 inhibitors can significantly alleviate the decrease in Rhodopsin expression induced by blue light irradiation in the retina. Inhibiting PARP-1 can inhibit mitochondrial autophagy and have a synergistic protective effect on retinal photodamage	661W cells; PARP-1 knockdown cell line	—	Zhang (2023a), Zhang (2023b)

## 5 Future perspectives

With the popularization of electronic equipment, the incidence rate of eye blue light injury is expected to continue to rise, especially among young and elderly people. It is predicted that by 2050, the incidence rate of eye diseases such as myopia, diabetes retinopathy, macular degeneration and glaucoma will increase significantly (Antemie et al., 2023; Landreneau et al., 2021). Therefore, future research directions may focus more on the role of blue light damage in ophthalmic diseases in young and elderly people. In the field of ocular pathophysiology, research will utilize more advanced imaging techniques to achieve real-time monitoring of the damage process of blue light to ocular tissues. The study may reveal new associations between blue light damage and genetics, environment, and lifestyle, and explore how these factors collectively affect eye health. Experimental modeling will focus more on simulating the actual human exposure to blue light, including factors such as light intensity, wavelength, and duration. It is also possible to develop models that are closer to human pathophysiological characteristics, such as using gene editing techniques to create animal models with specific genetic backgrounds.

Although some drugs and supplements have been used to treat blue light damage, their efficacy and safety still need further validation. Natural medicine has unique advantages in treating BLED due to its multi-component synergistic effect, overall regulation, and mild and safe characteristics. Therefore, it is necessary to explore the mechanism of action of natural medicine components in preventing and treating blue light damage, as well as how to integrate them with modern medicine. Meanwhile, delving deeper into the molecular mechanisms of blue light damage can help discover new therapeutic targets. At present, RNA nanomedicine has been used to achieve specific targets in the clinical treatment of eye diseases (Zhang et al., 2024). In order to improve the therapeutic effect, it is necessary to develop new drugs and formulations to enhance the permeability and bioavailability of drugs in the eye. It is pointed out that hydrogels can prolong the residence time of drugs in the eye and control drug release, thus improving the bioavailability of drugs (Han et al., 2024). These research findings provide new directions for future drug development.

In addition, the ocular microbiome is a dynamic ecosystem that plays a crucial but yet to be fully explored role in human health. The immune pardon mechanism of the eye includes the blood aqueous barrier, blood retinal barrier, and anti-inflammatory cytokines, all of which are important components. Recent studies have begun to explore the role of the ocular surface in the lung eye axis, emphasizing the bidirectional relationship between the respiratory system and eye health, suggesting that respiratory diseases may have an impact on eye diseases (Allam et al., 2024). Other studies have shown that gut microbiota is not only involved in the occurrence and development of various extraintestinal diseases, but also closely related to ophthalmic diseases such as uveitis, age-related macular degeneration, and glaucoma. The concept of gut eye axis can be used to explain the impact of gut microbiota imbalance on eye health (Zhang H. et al., 2023; Napolitano et al., 2021). Although research on the gut eye axis is currently insufficient and controversial, and cannot fully explain all existing issues, elucidating the pathological mechanisms related to gut microbiota and common eye diseases may provide new ideas for disease diagnosis and treatment.
